# Nasal septal abscess diagnosed by ultrasound

**DOI:** 10.1186/2036-7902-6-S1-A31

**Published:** 2014-01-31

**Authors:** Mingtse Tsai, Jengtan Sun, Kuangchau Tsai, Wanching Lien

**Affiliations:** 1 Department of Emergency Medicine, Far Eastern Memorial Hospital, New Taipei City, Taiwan; 2 Department of Emergency Medicine, National Taiwan University Hospital, Taipei, Taiwan

## 

A 77-year-old healthy woman was referred to our hospital due to nasal pain for 2 weeks. She denied nasal trauma in recent months. The local physical finding showed a bulging nasal septum. Ultrasonography applied transversely on the patient’s nose showed a heterogeneous 1-centimeter-in-diameter mass, which was further confirmed by computed tomography. The abscess was drained and the bacteriological cultures revealed viridans streptococci. Follow-up ultrasonography 2 weeks later showed nostrils without septal mass.

Nasal septal abscess is uncommon. Usually it is secondary to nasal trauma. There are other less common causes like sinusitis or dental infections.1 Immunocompromised status should be considered if there is no history of nasal trauma.2 If left untreated, there are risks of intracranial complications and facial deformity. The reported pathogens include bacteria and fungi, among which viridans streptococci are the most common. Proper management requires prompt diagnosis, adequate surgical drainage, and antibiotics to prevent the potentially dangerous spread of infection and the development of severe functional and cosmetic sequelae.

Ultrasonography was regarded as an efficient diagnositic tool in practice currently. In the field of otorhinolaryngology, it is applied in several diseases such as sinusitis, peritonsillar abscess or lymphadenitis. With ultrasonography, physicians can assess this disease quickly and treat it promptly.

